# Draft Genome Sequence of Komagataeibacter maltaceti LMG 1529^T^, a Vinegar-Producing Acetic Acid Bacterium Isolated from Malt Vinegar Brewery Acetifiers

**DOI:** 10.1128/genomeA.00330-18

**Published:** 2018-04-19

**Authors:** Qingyang Zhang, Anja Poehlein, Jacqueline Hollensteiner, Rolf Daniel

**Affiliations:** a Genomic and Applied Microbiology and Göttingen Genomics Laboratory, Institute of Microbiology and Genetics, University of Göttingen, Göttingen, Germany

## Abstract

We present the genome sequence of Komagataeibacter maltaceti LMG 1529^T^, which is a vinegar-producing acetic acid bacterium. The draft genome sequence consists of 3.6 Mb and contains 3,225 predicted protein-encoding genes. In addition, 53 genes encoding potential oxidoreductases were identified.

## GENOME ANNOUNCEMENT

The type strain Komagataeibacter maltaceti LMG 1529 (formerly Gluconacetobacter maltaceti) is a vinegar-producing acetic acid bacterium which was first isolated from malt vinegar brewery acetifiers in 1956 (1). Acetic acid bacteria are obligate aerobes and are well known for their acetic acid production by alcohol dehydrogenases (2–4). Several strains are used for traditional vinegar production (2, 5–7). Previous studies showed that most of these alcohol dehydrogenases exhibit a broad substrate spectrum, including primary, secondary, aliphatic, and aromatic alcohols, which can be used for chiral building blocks in industry (8–11). In addition, acetic acid bacteria are used as biocatalysts in pharmaceutical and cosmetic industries (12, 13). Some acetic acid bacteria are cellulose producers, including the species Komagataeibacter xylinus (3, 14). To provide insights into the metabolic and biocatalytic potential of K. maltaceti LMG 1529^T^, the whole genome was sequenced and analyzed.

The genomic DNA of K. maltaceti LMG 1529^T^ was extracted by using the MasterPure complete DNA purification kit, as recommended by the manufacturer (Epicentre, Illumina, Madison, WI, USA). The isolated DNA was used to generate Illumina shotgun paired-end sequencing libraries. The MiSeq system and the MiSeq reagent kit version 3 were used for sequencing and applied as recommended by the manufacturer (Illumina, San Diego, CA, USA). The resulting reads were quality filtered using Trimmomatic version 0.36 (15). A total of 3,695,976 paired-end reads were obtained. The SPAdes genome assembler version 3.11.0 (16) was used to perform a *de novo* genome assembly, which yielded 163 contigs (>500 bp) and 202-fold coverage. The assembly was validated with QualiMap version 2.1 (17).

The draft genome sequence consists of 3,629,663 bp, with an overall G+C content of 59.14%. The genome annotation was performed with Rapid Prokaryotic Genome Annotation (Prokka) tool version 1.11 (18). The predicted 3,281 genes included 49 tRNA genes, 6 rRNA genes, 1 transfer-messenger RNA (tmRNA) gene, and 3,225 protein-encoding genes, of which 1,740 had functional predictions. The phylogenetic relationships to closest relatives, including Komagataeibacter medellinensis NBRC 3288 (14), were determined. Classification was performed by calculating the average nucleotide identity with the Python module for average nucleotide identity analyses (pyANI) version 0.2.7 (19). This analysis revealed that the K. maltaceti type strain LMG 1529 represents its own species group within the genus, as less than 94% nucleotide identity to other type strains of the genus was recorded.

Genome analysis revealed that strain LMG 1529^T^ harbors the potential to produce cellulose. Especially, the deduced proteins KMAL_17180, KMAL_17160, and KMAL_17170 exhibited high sequence identity (89%, 95%, and 97%, respectively) to AscAB, AscC, and AscD of the cellulose synthesis-encoding operon of Gluconacetobacter hansenii ATCC 53582 (20, 21). In addition, 53 genes encode potential oxidoreductases, including 3 alcohol dehydrogenases and 9 short-chain dehydrogenases/reductases. Members of the short-chain dehydrogenase/reductase superfamily of oxidoreductases are interesting candidates in green chemistry for the conversion of bulky substrates.

### Accession number(s).

The whole-genome shotgun project of Komagataeibacter maltaceti LMG 1529^T^ has been deposited at DDBJ/ENA/GenBank under the accession number POTC00000000. The version described in this paper is version POTC01000000.
